# Efficient Identification of Assembly Neurons within Massively Parallel Spike Trains

**DOI:** 10.1155/2010/439648

**Published:** 2009-09-29

**Authors:** Denise Berger, Christian Borgelt, Sebastien Louis, Abigail Morrison, Sonja Grün

**Affiliations:** ^1^Bernstein Center for Computational Neuroscience, Humboldt-Universität zu Berlin, 10115 Berlin, Germany; ^2^Neuroinformatics, Institute of Biology, Department of Biology, Chemistry, and Pharmacy, Freie Universität, 14195 Berlin, Germany; ^3^European Centre for Soft Computing, 33600 Mieres, Asturias, Spain; ^4^RIKEN Brain Science Institute, Wako-shi 351-0198, Japan

## Abstract

The chance of detecting assembly activity is expected to increase if the spiking activities of large numbers of neurons are recorded simultaneously. Although such massively parallel recordings are now becoming available, methods able to analyze such data for spike correlation are still rare, as a combinatorial explosion often makes it infeasible to extend methods developed for smaller data sets. By evaluating pattern complexity distributions the existence of correlated groups can be detected, but their member neurons cannot be identified. In this contribution, we present approaches to actually identify the individual neurons involved in assemblies. Our results may complement other methods and also provide a way to reduce data sets to the “relevant” neurons, thus allowing us to carry out a refined analysis of the detailed correlation structure due to reduced computation time.

## 1. Introduction

Synchronized presynaptic spiking activity is known to have a higher efficacy in generating output spikes than noncoordinated spike timing [1]. Therefore, temporal coordination of spike timing is a commonly accepted signature of neuronal assembly activity [2–5]. Consequently, approaches to detect assembly activity have focused on the detection of correlated spiking activity on a millisecond time resolution. 

 With massively parallel recordings becoming available at an accelerating rate [6], the likelihood of observing the signature of assembly activity is improving. However, we still lack the corresponding analysis tools [7]. Most of the existing methods are based on pairwise analysis, for example, [8–10]. Approaches to analyze correlations between more than two neurons exist, but typically only work for a small number of neurons [11–15] or only consider pairwise correlations when analyzing the assembly [16–19] (in these approaches a set of neurons is seen as an assembly if most of them are pairwise correlated). 

 It is usually infeasible to simply extend existing methods that identify individual spike patterns to massively parallel data due to a combinatorial explosion. Therefore, in previous studies, we tried new approaches that evaluate the complexity distribution [20, 21] or the intersection matrix [22], which can handle massively parallel data in reasonable computational time and analyze it for higher-order spike patterns. These methods are able to detect the presence of higher-order correlation, but do not identify neurons that participate in the correlation. The goal of the present study is to resolve this issue: we want to directly identify neurons that take part in an assembly as expressed by coincident firing. Our aim is not, however, to determine the order of the correlation in which they are involved, but to provide an efficient tool to reduce the dataset to the relevant neurons, which can then be examined in detail in further analysis. We present two different methods, both of which rely on the idea of detecting whether an individual neuron is involved in any kind of coincident event more often than can be expected by chance. 

 The paper is organized as follows: in Section 2 we discuss methods of generating surrogate data from given spike trains, which we need in order to obtain reference distributions for the test statistics that are introduced in Section 3. In Section 4 we apply our test statistics to several artificial and one real-world dataset and assess their performance. Finally, in Section 5 we evaluate our findings and draw conclusions about the usefulness of our approach. This study is based on a former contribution [23], and is extended here by a systematic study of parameter dependencies and the analysis of simulated network data and neuronal data.

## 2. Generation of Surrogate Data

Our methods of detecting neurons that are participating in an assembly consist of two ingredients: a test statistic (described in the following section) and a procedure to generate surrogate data (described in the this section), which is needed to estimate their distribution. Starting with the general surrogate generation procedure, we discuss common problems and examine two concrete approaches.

### 2.1. General Procedure

In all approaches explored in this paper, we compute a different test statistic from the data, each of which is based on a different basic idea (see Section 3). Unfortunately, there are certain obstacles that prevent us from easily finding the distributions of these test statistics under the null hypothesis that the considered neuron *i* is not part of an assembly. Therefore, we rely on the generation of * surrogate data* from the original dataset in order to estimate this distribution. The surrogate dataset is created in such a way that a neuron *i* under consideration, if it is part of an assembly, becomes independent of all other neurons, or at least is considerably less dependent on the other neurons than in the original dataset. 

 The general test procedure is as follows: first we compute, for the neuron *i* under consideration, the test statistic on the original dataset. Then we generate a surrogate dataset in one of the ways described in what follows, recompute the test statistic, and compare the result to the result obtained on the original dataset. Generating surrogate datasets and recomputing the test statistic is repeated sufficiently often (unless otherwise stated, 5000 times). Finally, counting the number of times the result of a surrogate run meets or exceeds the result obtained on the original data and dividing this number by the total number of runs yields a *P*-value. This *P*-value measures the probability that a result as obtained on the actual data would be obtained by chance if neuron *i* was independent of all other neurons (where what exactly is meant by “chance” is made precise by the surrogate generation method).

### 2.2. Problems of Generating Surrogate Data

In general, the procedure with which surrogate data is generated is critical for the validity of a test for assembly activity, because it implicitly represents the null hypothesis. As a consequence, a common argument against a chosen surrogate generation method is that it represents a (much) too restricted null hypothesis. In such a case obtaining a significant test result could be explained not only by the original data actually having the property tested for but also by some other, unrelated property, which should have been covered by the null hypothesis, but was not. In principle, the null hypothesis must cover the full complement of the property tested for (which, of course, represents the alternative hypothesis). The “worst” data model in this complement—that is, the data model that is most likely to produce, by chance, data that looks like as if it was generated by a model having the property tested for—determines the significance of a test result. 

 A closer analysis reveals that this problem cannot in general be fully overcome for a test for assembly activity, because the complement of assembly activity is too large. In this complement there are data models that are so close to models exhibiting assembly activity (i.e., models that can, with a high probability, generate the same data), that it is almost impossible to obtain significant *P*-values. In order to cope with this problem, one has to restrict the null hypothesis. However, at the same time one must take care to restrict it only to such an extent that the restrictions can reasonably be considered harmless, where “harmless” means that only such models are excluded of which we are fairly certain that they are impossible. 

 In practice, it is this idea that underlies the desire to preserve, as much as possible, certain properties of the original data, which are not directly connected to the property tested for, but could explain a significant test result. Unfortunately, there is no consensus about which properties should or must be preserved. Examples include the overall and local firing rates of the neurons, the distribution of inter-spike intervals, the number of spikes per time bin and so forth. For an extensive discussion of this problem see [24]. 

 In addition, the more properties one tries to preserve, the more complex and computationally more expensive generating surrogate datasets becomes. If one recalls that a large number of surrogate datasets have to be generated in order to be able to compute a sufficiently small and reliable *P*-value, the required computation time is a serious concern. 

 As a consequence, we confine ourselves here to fairly simple methods of generating surrogate data with the following argument: we see our method mainly as a preprocessing method and always assume that the findings of our methods are later substantiated with more detailed and statistically more powerful tests. Our primary goal is to reduce the number of neurons that have to be subjected to this more detailed and thus also computationally more costly analysis. Therefore, we can afford to have a few false-positive results, because it can reasonably be assumed that they will be detected as such in subsequent steps. However, we cannot tolerate false negatives, since then neurons that are actually relevant would be excluded from further analysis. Relying on an overly restricted null hypothesis, implemented as an over-simplified surrogate generation method that does not preserve all relevant properties of the original data, may produce additional false-positives but will not produce false negatives. For the purposes of preprocessing, we therefore prioritize speed over accuracy and use simple surrogate data generation methods.

### 2.3. Spike Shuffling in Time

One of the simplest and fastest ways of generating a surrogate dataset in which a considered neuron *i* is independent of all other neurons is to shuffle the spike times of neuron *i*. That is, the spikes of neuron *i* are reassigned from their original time bins to time bins that are randomly chosen from the available ones. Note that we shuffle * only* the spikes of neuron *i*; all other spikes are left unchanged. As a consequence, the correlation structure between all other neurons is preserved—only correlations of neuron *i* with other neurons are destroyed. 

 Some obvious advantages of such a shuffling scheme are that it preserves the number of spikes of the considered neuron, changes the number of spikes per time bin by at most 1 (only a spike of the considered neuron can be removed or added), is easy to program, and is fast to execute. However, it has the serious disadvantage that in real-world data firing rates are not constant over time. For a thorough discussion of this issue see [24]. 

 If we assume that the neurons under consideration change their firing rates coherently, that is, the firing rate rises or falls for all neurons in parallel, there is a fairly simple, but nevertheless surprisingly effective approach to cope with varying spike rates: we simply see the number of spikes in a bin as a very coarse indicator of the general neuronal activity level. We may then derive an estimate for the probability of a spike occurring in a time bin *j* as follows: let *I*_*l*_ be the index set of the neurons that fire in the *l*th time bin, and let *T* be the total number of time bins. Then we may define the probability of randomly assigning a spike of some neuron *i* to a given time bin *j* as

(1) $$\begin{array}{c} \pi_{j}=\frac{\left|I_{j}\right|+c}{\sum_{l=1}^{T}\left(\left|I_{l}\right|+c\right)}, \end{array}$$

where *c* is a correction term that in similar contexts is known as the * Laplace correction*. Formally, this correction term can be justified with arguments from Bayesian statistics [25], where it is derived from an uninformative prior distribution. This prior distribution is a uniform distribution here, because formally we consider a polynomial distribution over the time bins. This prior distribution is then modified with the data distribution using Bayes' rule. As a consequence, the Laplace correction *c* introduces a tendency toward a uniform distribution and may be chosen in [0, +∞). For *c* = 0 the number of spikes in a bin relative to the total number of spikes directly determines the probability of assigning a spike to this time bin. This yields a maximum likelihood estimation of the time bin probability, because no weight is assigned to the uniform prior distribution; one relies entirely on the available data. For *c* → ∞ we obtain, as a limiting case, the uniform spike shuffling scheme described above: each time bin has the same probability of having a spike assigned to it, regardless of the number of spikes it contains. Values 0 < *c* < ∞ introduce a limited tendency toward a uniform distribution, which becomes stronger for greater values of *c*.

### 2.4. Trial Shuffling

In real recordings of parallel spike trains it is often the case that some kind of stimulus is presented to the subject and then the neuronal response to this stimulus is recorded for a certain period. In order to reduce the effects of random influences that may be present in a single instance, several *trials* of presenting the stimulus and recording the response are carried out. 

 If multiple trials are available, one may use * trial shuffling* to generate surrogate data. In trial shuffling the spike trains of a neuron *i* under consideration are randomly assigned to other trials, thereby replacing the original spike trains in the respective trials. Even though all trials must, of course, be aligned w.r.t. the onset of the stimulus, the individual spike trains vary sufficiently due to their stochastic nature, such that neuron *i* becomes independent of the other neurons in terms of the precise timing of spikes across the neurons. At least it is plausible to assume that existing dependencies will be considerably reduced due to the independence of the trials. 

 However, one should be aware that because of similar responses due to the same stimulus, trial shuffling can hide assembly activity that is actually present, despite the independence of the trials. In particular this is the case if the assembly response is sufficiently exact in time relative to the stimulus onset, so that trial shuffling does not destroy the dependence between the neurons. As a consequence, trial shuffling can lead to false negatives, which is a critical disadvantage for our approach (see Section 2.2). 

 In addition, plain trial shuffling has the disadvantage that the number of available trials limits the achievable *P*-values: suppose there are *s* trials. Then one is the original data and the spike train of the neuron *i* under consideration can be moved to at most *s* − 1 other trials in order to obtain surrogate data. Even if in all of these *s* − 1 cases the obtained statistic value is less than that obtained on the original data, all we can say about the *P*-value is that it is less than 1/(*s* − 1). Since *s* usually ranges only in the order of a few dozen, it may not even be possible to obtain a significant test result. The fact that we can use any trial as the original data and all other trials to generate surrogates does not amend this drawback, because this only provides us with *s* independent *P*-values (cf. [26]). 

 In order to improve this situation, we concatenate the individual trials into one long dataset and compute the test statistic once on the entire set. Then the trial segments of the spike train of the neuron *i* under consideration are shuffled to obtain a surrogate dataset. As a consequence, the number of surrogate datasets that can be generated is considerably increased, because any permutation of the trial segments of the spike train (except the identity, that is, the case in which all trial segments are at their original positions) yields a surrogate dataset. 

 However, one should be aware that this scheme is also open to criticism: suppose that two different permutations move a given trial segment into the same location. Then the terms of the test statistic that result from the time bins in the corresponding trial are necessarily the same for both permutations. This may have the effect that the obtained distribution of the test statistic exhibits less variation than the actual distribution. If avoiding this effect is made a strict requirement, only *s* − 1 permutations (other than the identity) can be defined. In this case there is no advantage over separate trials. 

 Nevertheless we believe that such a trial shuffling scheme is reasonable. In the first place, the fraction of terms of the test statistic that are identical for two permutations will be relatively small unless the total number *s* of trials is very small. Hence the effect on the variability of the test statistic is likely negligible. Secondly, reducing the variability of the test statistic for surrogate data has at most the effect of creating false-positive results. As already argued in Section 2.2, we can afford a few false-positive results, because later processing steps should remove them.

## 3. Test Statistics

We explored the performance of two approaches to identify whether a neuron is part of a correlated group of neurons.

### 3.1. Conditional Pattern Complexities (CPCs)

The first approach to identify neurons participating in assemblies is based on the idea that such neurons should have, on average, more neurons firing together with them in the original data than in the surrogates. In other words, if some neuron *i* participates in one or more large assemblies, there should be several time bins in which it fires together with a larger number of other neurons. Hence the average complexity of patterns involving neuron *i* should be larger than can be expected by chance. Formally, we use

(2) $$\begin{array}{c} \begin{array}{c} \overset{̅}{\mu }\left(i\right)=\frac{1}{T}\overset{T}{\underset{l=1}{\sum }}\left|I_{l}-\left\{i\right\}\right|, \end{array}\\ \mu \left(i\right)=\frac{1}{T_{i}}\overset{T}{\underset{l=1}{\sum }}1_{I_{l}}\left(i\right)\left|I_{l}-\left\{i\right\}\right|, \end{array}$$

where *I*_*l*_ is the index set of the neurons that fire in the *l*th time bin, **1**_*I*_*l*__(*i*) is the indicator function of the set *I*_*l*_ (which is 1 if *i* ∈ *I*_*l*_ and 0 otherwise), *T* is the total number of time bins, and *T*_*i*_ = ∑_*l* = 1_^*T*^ **1**_*I*_*l*__(*i*) is the number of time bins in which neuron *i* fires. Thus, ${\overset{̅}{\mu }}_{i}$ is simply the overall average pattern complexity with spikes of neuron *i* removed, while *μ*_*i*_ is the average pattern complexity in those time bins in which neuron *i* fires, again with spikes of neuron *i* removed. We may also call this the conditional average pattern complexity, that is, conditional on spikes of neuron *i*. A natural test statistic is

(3) $$\begin{array}{c} t^{\text{CPC}}\left(i\right)=\frac{\mu \left(i\right)-\overset{̅}{\mu }\left(i\right)}{\overset{̅}{\mu }\left(i\right)}. \end{array}$$

A *P*-value can be derived by using the surrogate data generation procedures described in Section 2. 

 An obvious way to improve this measure is to weight large complexities more strongly than smaller ones, because large complexities are, intuitively, more indicative of assembly activity. A simple technical means to achieve such weighting is to raise the complexities to a user-specified power *α*:

(4) $$\begin{array}{c} \begin{array}{c} {\overset{̅}{\mu }}_{\alpha }\left(i\right)=\frac{1}{T}\overset{T}{\underset{l=1}{\sum }}{\left|I_{l}-\left\{i\right\}\right|}^{\alpha }, \end{array}\\ \mu_{\alpha }\left(i\right)=\frac{1}{T_{i}}\overset{T}{\underset{l=1}{\sum }}1_{I_{l}}\left(i\right){\left|I_{l}-\{i\}\right|}^{\alpha }. \end{array}$$

In other words, instead of a simple mean of the pattern complexities, we employ higher moments. The resulting test statistic is

(5) $$\begin{array}{c} t_{\alpha }^{\text{CPC}}\left(i\right)=\frac{\mu_{\alpha }\left(i\right)-{\overset{̅}{\mu }}_{\alpha }\left(i\right)}{{\overset{̅}{\mu }}_{\alpha }\left(i\right)}. \end{array}$$

### 3.2. Conditional Spike Frequencies (CSFs)

In a second approach we take into account how often other individual neurons fire together with neuron *i*. The idea is that if neuron *i* participates in one or more assemblies, it should fire more often together with certain other neurons, specifically those also in the assemblies, than can be expected by chance. In order to be less sensitive to differing firing rates, we use the number of excess spikes to form a test statistic: we compute for each neuron *j*, *j* ≠ *i*, the difference between the actual number *T*_*ij*_ = ∑_*l* = 1_^*T*^ **1**_*I*_*l*__(*i*)**1**_*I*_*l*__(*j*) of spikes of neuron *j* observed together with a spike of neuron *i* and the expected number of such spikes, estimated as $T_{i}{\hat{\eta }}_{j}$ with ${\hat{\eta }}_{j}=T_{j}/T$, where *T*_*i*_ = ∑_*l* = 1_^*T*^ **1**_*I*_*l*__(*i*) and *T*_*j*_ = ∑_*l* = 1_^*T*^ **1**_*I*_*l*__(*l*) are the number of time bins in which neurons *i* and *j*, respectively, fire and *T* is the total number of time bins. Since only excess spikes tell us about possible correlations, negative differences are ignored. Formally, the test statistic is

(6) $$\begin{array}{c} t^{\text{CSF}}\left(i\right)=\frac{1}{N-1}\overset{N}{\underset{j=1,j\neq i}{\sum }}\zeta \left(T_{ij}>T_{i}{\hat{\eta }}_{j}\right)\left(T_{ij}-T_{i}{\hat{\eta }}_{j}\right), \end{array}$$

where *N* is the total number of neurons and *ζ*(*φ*) is 1 if *φ* is true and 0 otherwise. Analogously to the conditional pattern complexity described above, we may consider weighting a large number of excess spikes more strongly than a small number, as a large number is certainly more indicative of assembly activity. In order to achieve this, we once again introduce a user-specified power *α* to which the number of excess spikes is raised:

(7) $$\begin{array}{c} t_{\alpha }^{\text{CSF}}\left(i\right)=\frac{1}{N-1}\overset{N}{\underset{j=1,j\neq i}{\sum }}\zeta \left(T_{ij}>T_{i}{\hat{\eta }}_{j}\right){\left(T_{ij}-T_{i}{\hat{\eta }}_{j}\right)}^{\alpha }. \end{array}$$

As another straightforward variant, one may relate the excess spikes to the expected number of spikes, because a higher firing rate also leads to a higher variation in the actual number of observed spikes and thus makes a large number of excess spikes more likely. In this case, the measure reads (with the optional power *α* already added)

(8) $$\begin{array}{c} {\tilde{t}}_{\alpha }^{\text{ CSF}}\left(i\right)=\frac{1}{N-1}\overset{N}{\underset{j=1,j\neq i}{\sum }}\zeta \left(T_{ij}>T_{i}{\hat{\eta }}_{j}\right){\left(\frac{\left(T_{ij}-T_{i}{\hat{\eta }}_{j}\right)}{T_{i}{\hat{\eta }}_{j}}\right)}^{\alpha }. \end{array}$$

However, it is not immediately clear whether this modification improves or deteriorates the sensitivity of this measure. In this paper we confine our study to the unmodified version. 

 A *P*-value is derived by the same surrogate generation procedures described earlier (see Section 2).

## 4. Results for Different Test Data Sets

We tested our statistics in three scenarios: data generated using the stochastic model that specifies the coordinated activity we desire to detect (Sections 4.1 and 4.2), data generated by simulating a large network of neurons into which a synfire chain is embedded (Sections 4.3 and 4.4) and real spike train recordings from cat visual cortex (Section 4.5).

### 4.1. Stochastic Model of Assembly Activity

The model of coordinated neural activity we adopt here has its origins in [21, 27, 28]. The basic assumption is that the activation of an assembly is expressed by synchronous spiking of its member neurons. Due to the typically blind sampling from the cortical tissue, the chance of observing a number of neurons from one assembly is rather small. This enters our modeling by assuming that only a small percentage of neurons are correlated—the rest fire independently. 

**Figure 1:**
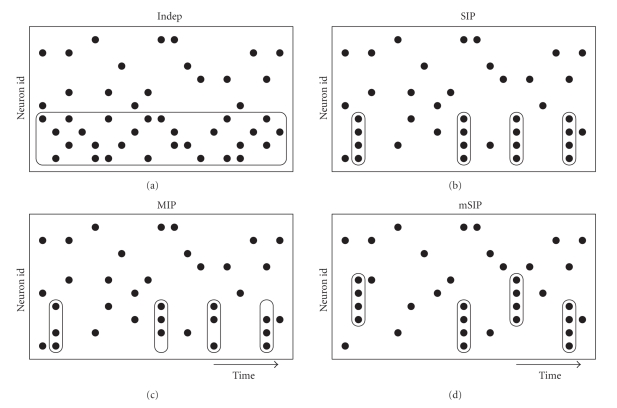
Sketches of different settings of our stochastic model. Indep: independent spike trains, but differing firing rates; SIP: single assembly, single interaction process; MIP: single assembly, multiple interaction process; mSIP: multiple assemblies, single interaction processes.

 In our correlation model simultaneous spike trains are modeled as parallel, binary processes realized as stationary Bernoulli processes. The simplest form realizes fully independent processes with predefined firing rates *λ*_*i*_ per neuron *i*, thus defining the occupation probability *p*_*i*_ = *λ*_*i*_ · *h* per time bin of length *h* for each process. Such realizations model the basic activity of the *N* neurons. Without further insertion of correlated spiking such independent spike trains serve as the control setting (“rate model”, labeled “Indep” in Figure 1).

Assembly activity is modeled by coincident spiking activity in a subset of *M* out of the *N* neurons: a hidden “mother” process of rate *λ*_*c*_ is realized, from which spikes are copied into the *M* selected child processes with probability *ϵ*. If the probability is 1 all *M* processes receive a copy of each spike of the hidden process. In this case all *M* neurons exhibit coincidences of order *M* (labeled “SIP” in Figure 1). A process of this type is also called a * single interaction process* (SIP) [28]. 

 Alternatively, and presumably more realistically for experimental data, the copy probability can be *ϵ* < 1. In this case the resulting coincidences within the *M* neurons are composed of *ϵ* · *M* < *M* spikes on average, with a random composition of spiking neurons per event (labeled “MIP” in Figure 1). A process of this type is also called a * multiple interaction process* (MIP) [28]. 

 Finally, the correlated and uncorrelated spike trains are merged. The spike train of a child process is then composed of “background” firing and of spikes involved in coincidences. The total firing rate is *λ*_*i*_ = *λ*_*b*,*i*_ + *λ*_*c*,*i*_, where *λ*_*b*,*i*_ is the background rate and

(9) $$\begin{array}{c} \lambda_{c,i}=\lambda_{c}\cdot \epsilon \end{array}$$

the coincidence rate. Trivially, the firing rates of each process can be predefined, and the background firing rate can be adjusted accordingly. 

 Multiple assemblies of neurons can be generated analogously by using one hidden process per assembly. The sets of neurons to which the spikes are copied from each of these processes may overlap or not. The total rate of neurons that take part in more than one assembly is composed of the sum of the assembly coincidence rates and the background rate: *λ*_*i*_ = *λ*_*b*,*i*_ + ∑_*j* = 1_^*m*^ *λ*_*c*,*ij*_ with assembly index *j*, 1 ≤ *j* ≤ *m*, where *m* is the number of assemblies (labeled “mSIP” in Figure 1). Note that multiple assemblies can be modeled—just like single assemblies—with a copy probability *ϵ* = 1 or with *ϵ* < 1. In the latter case, each assembly may even use a different copy probability *ϵ*_*j*_, so that we have *λ*_*c*,*ij*_ = *λ*_*c*,*j*_ · *ϵ*_*j*_, where *λ*_*c*,*j*_ is the firing rate of the *j*th assembly and *ϵ*_*j*_ the corresponding copy probability. 

 These stochastic models (with no groups, one group, and several groups of correlated neurons) specify what we understand by the assembly activity we desire to detect. Of course, these models are not exhaustive in capturing what may be understood by “assembly activity”, but merely define the focus of our study.

### 4.2. Results for the Stochastic Model Data

#### 4.2.1. Test Statistic per Neuron

In a first experiment to demonstrate our approaches we make use of datasets generated by the stochastic model (Section 4.1). In these datasets we have full control over the statistics of the spike trains (stationary Poisson) and the correlation structure between the neurons. Thus, the analyses of these datasets serve as a proof of principle. 

**Figure 2:**
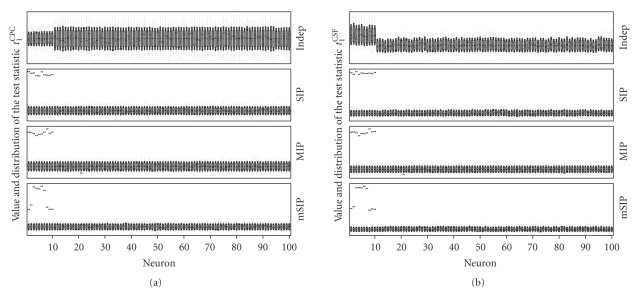
Analysis results for datasets representing the settings Indep, SIP, MIP, and mSIP using *t*_1_^CPC^ (a) and *t*_1_^CSF^ (b). All sets are composed of *N* = 100 neurons. In Indep, neurons 1–10 (*M* = 10) have a higher firing rate (*λ* = 50 Hz) than the rest of the neurons (*λ* = 20 Hz). In SIP, MIP and mSIP the first *M* = 10 neurons are involved in synchronous events, the rest are independent. All neurons have the same firing rate, *λ* = 20 Hz. The coincidence firing rate of the assemblies is *λ*_*c*_ = 5 Hz. The synchronous events in MIP are generated with a copy probability of *ϵ* = 0.8. In each panel, box plots show the distribution (wide box: 5% to 95%, narrow box: 1% to 99%, whiskers: minimum to maximum) of the shuffling results (*s* = 10^5^ runs) for each neuron *i* (ids ordered along the *x*-axis). Beyond the extent of the whiskers, results are significant on a 1/*s* level. The test statistic values *t*_1_^CPC^ and *t*_1_^CSF^ obtained on the actual data are shown as bow-ties.

Figure 2 shows results from the four datasets analyzed using the test statistics *t* ^CPC^ Figure 2(a) and *t* ^CSF^ Figure 2(b). The datasets are composed of *N* = 100 parallel spike trains of which the first *M* = 10 neurons either have a different firing rate then the rest of the neurons (“Indep”) or are correlated via synchronous spike events (“SIP”, “MIP”, “mSIP”). Each diagram shows the value of the test statistic (bow-tie) and a box plot indicating the distribution of the surrogate data results (spike shuffling in time) for each of the 100 neurons (wide boxes: 5%-quantile to 95%-quantile, narrow boxes: 1%-quantile to 99%-quantile, whiskers: minimum to maximum statistics value obtained in the shuffling runs). The spike shuffling procedure was chosen as uniform, because the data are generated as stationary Poisson processes. 

 For “Indep” the tests do not produce a significant result for any of the neurons, since they are independent. For the other three datasets (“SIP”, “MIP”, “mSIP”) the tests detect that the first 10 neurons have excess coincidences, thus exactly identifying the neurons involved in assemblies. Significance is highest for “SIP”, due to the fact that all correlated neurons participate with probability *ϵ* = 1 in the coincident events. Significance is slightly reduced for “MIP”, but all neurons participating in the assembly are still reliably detected. 

 For the mSIP setting all neurons taking part in assemblies are also reliably detected. Significance is higher for the neurons participating in both assemblies as compared to the ones participating in only one of the assemblies.

#### 4.2.2. False Positive and False Negative Rate as a Function of Assembly Size

In the following we investigate how assembly size affects the quality of detection in two of the four settings identified in Section 4.1, namely SIP and MIP. In addition, we make use of the additional parameter *α* introduced in Section 3 and explore *α* = 1 (default, standard form of the measures) and *α* = 3 for both test statistics *t*_*α*_^CPC^ and *t*_*α*_^CSF^, and thus consider a total of four measures. 

**Figure 3:**
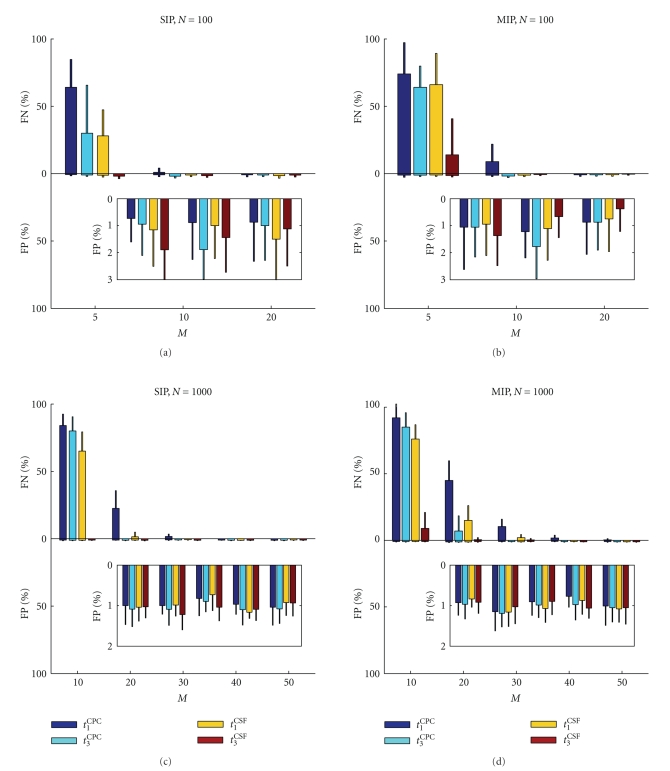
False positive and false negative rate as a function of assembly size *M*. Each panel shows the FN rate as bar plots (positive *y*-axis) and FP rate (negative *y*-axis, and inset) for each type of dataset (a): SIP, *N* = 100; (b) MIP, *N* = 100, *ϵ* = 0.8; (c) SIP, *N* = 1000; (d) MIP, *N* = 1000, *ϵ* = 0.8. Each panel contains a sets of grouped bars (colors indicate the results for the different test statistics *t*_1_^CPC^, *t*_3_^CPC^, *t*_1_^CPC^, *t*_3_^CSF^; see legend) for each *M*. Each bar represents the mean of 10 realizations, and the error bar indicates one standard deviation. All datasets have stationary firing rates (all neurons *λ* = 20 Hz), a stationary coincidence rate of *λ*_*c*_ = 1 Hz, and a duration of *k* = 10 000 time steps of *h* = 1 milliseconds. Significance level is 1%. For generation of the surrogates (5000 repetitions), spikes are shuffled with uniform distribution in time.

Figure 3 depicts the false-positive (FP) and false negative (FN) rate for the four test statistics (indicated by different colors) as a function of assembly size *M* and total number of recorded neurons *N*. The FN rate is derived as the percentage of neurons that participate in assembly activity but are not detected (with a significance level of 1%; positive *y*-axis). For a low coincidence rate of *λ*_*c*_ = 1 Hz and a small number of neurons involved in the assembly (*M* = 5 for *N* = 100 for SIP and MIP, see Figures 3(a) and 3(b)) the percentage of FN is very high, in particular for MIP (close to 100%). Interestingly, for larger numbers of observed neurons (*N* = 1000, Figures 3(c)  and 3(d)) even for a smaller percentage of neurons involved in assemblies (*M* = 10, i.e., *M*/*N* = 10/1000 = 1/100 as compared to 5/100 in Figures 3(a)  and 3(b)) the percentage of FN is lower. If we consider the case that 5% of neurons are involved in assemblies (i.e., *M* = 5 for *N* = 100 and *M* = 50 for *N* = 1000), significant FNs can be observed for the smaller neuron set, whereas no FNs occur for the larger set. For the SIP cases, increasing the assembly size leads to a fast drop of the FNs, for MIP the decrease is more gradual. This is true for all four test statistics. The percentage of false-positives (Figure 3, negative *y*-axis), that is, neurons wrongly detected as assembly members, is very low in all cases, that is, at about the level expected by the chosen significance level of 1%. There is no obvious difference in the number of FPs generated between the different datasets or test statistics.

#### 4.2.3. Impact of Coincidence Rate on False Positives and False Negatives

**Figure 4:**
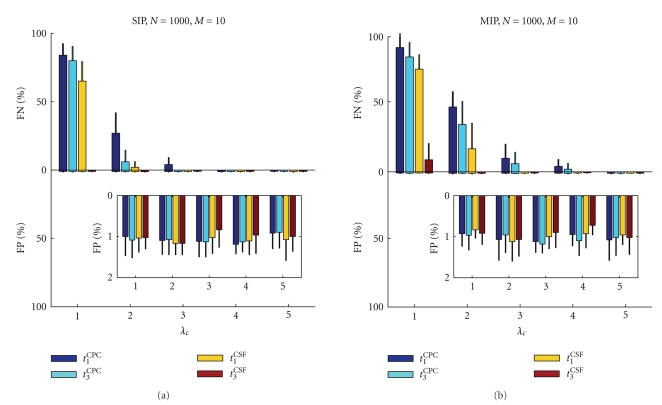
FP rate and FN rate as a function of the coincidence rate *λ*_*c*_ = 1, 2, 3, 4, 5 Hz shown for (a) SIP and (b) MIP with *N* = 1000 neurons and *M* = 10 assembly members. Display style and other parameters as in Figure 3.

In the previous paragraph we showed that the smaller the assembly size, the harder it is to detect all of its members. Here we demonstrate the dependence of the FP and FN rate as a function of the coincidence rate *λ*_*c*_ for a small assembly size (*M* = 10) to discuss a worst case scenario (see Figure 4). Here different test statistics lead to quite different results. *t*_3_^CSF^ achieves very good detection (low FN rate, FP ≈ 1%) for all coincidence rate levels for both SIP and MIP data. In contrast, the other test statistics result in a high FN rate (over 50%) for the SIP data with a small coincidence rate, but this drops to 0% as the coincidence rate increases from 1 to 2 Hz. Thus the presence of at least 2 Hz · 10 000 milliseconds = 20 SIP events leads to a perfect detection (Figure 4(a)). For *N* = 100 (not shown here) we found comparable results for increasing coincidence rate. In the case of MIP, the detection of assembly members becomes gradually better with increasing coincidence rate, and all assembly neurons are detected for *λ*_*c*_ ≥ 4 Hz (Figure 4(b)). The FNs decay faster for the *t*_*α*_^CSF^ test statistics than for the *t*_*α*_^CPC^ test statistics. 

 To gain insight into the excellent performance of *t*_3_^CSF^ for MIP, we will consider the impact of excess coincidences in the following example for the simpler case of *α* = 1. For a coincidence rate of *λ*_*c*_ = 1 Hz, approximately 10 MIP events are in the data. Due to the copy probability of *ϵ* = 0.8, on average only *M* · *ϵ* = 8 neurons of the assembly participate in a synchronized event. Within these 10 occurring synchronous events a specific pair of neurons has on average 0.8^2^ · 10 = 6.4 coincident spike events. To this we have to add ((*λ* − *λ*_*c*_) · *h*)^2^ · *T* = 3.61 coincidences resulting from background spikes. Thus we arrive at a total of approximately 10.1 coincidences. This exceeds by approximately 6.1 the expected number of (*λ* · *h*)^2^ · *T* = 4 coincidences of independently firing neurons. Since *t*_*α*_^CSF^ sums over all pairs neuron *i* has with other neurons, all *M* − 1 pairs containing the neurons of the assembly will contribute with this difference to the value of the *t* ^CSF^ statistic. 

 This can be expressed in a more formal way by considering expected values. *t* ^CSF^ takes the sum of the pairwise correlations between neuron *i* and all other neurons. For coincidences of neuron *i* ∈ {1,…, *M*} with neurons also in {1,…, *M*}, the contributions exceed the expected value and *M* − 1 terms add to the sum. Inserted coincidences occur at *T*_*c*_ = *T* *λ*_*c*_ times with probability *ϵ* ^2^. The rest of the spikes that occur outside of injected events contribute to chance coincidences with their background rate *λ* − *λ*_*c*_. The summands combining neuron *i* with the other *N* − *M* neurons each contribute a noise term *n*_*σ*_. This is approximated by

(10) $$\begin{array}{c} \frac{M-1}{N-1}\frac{T}{h}\left(\epsilon^{2}\lambda_{c}h+{\left(h(\lambda -\epsilon^{2}\lambda_{c})\right)}^{2}-\lambda^{2}h^{2}\right)+\frac{N-M}{N-1}n_{\sigma }. \end{array}$$

In contrast, if we consider a neuron that is not in {1,…, *M*}, the *M* − 1 terms now join the noise contributions and thus the above reduces to *n*_*σ*_. 

 Clearly, for a fixed number of neurons *N*, the larger the assembly size *M*, the more terms contribute with large coincidence counts, resulting in a larger significance and fewer FNs. For a fixed relation of *M*/*N* but increasing *N* expression (10) does not change. As a consequence, the expected difference between the *t* ^CSF^ value for an assembly neuron in the original data and its *t* ^CSF^ value after shuffling the spikes of this neuron remains the same in this case. However, the variance of the value of *t* ^CSF^ (for an independent neuron, and thus also for an assembly neuron after shuffling) decreases with increasing *N*, since it is an average of identically distributed summands. Hence the same expected difference should yield lower *P*-values and thus fewer false negatives. This is exactly what we observe in our experiments. 

 The coincidence rate *λ*_*c*_ enters directly as a factor in the term of the excess coincidences (first summand), and contributes only little to reducing the chance coincidences originating from background spikes (second summand). The expected number of coincidences (third summand) and the noise term are not affected by the injected coincidences. Thus increasing the coincidence rate has a strong effect on the resulting significance, and thereby indirectly reduces the FN rate. 

 The difference between the results for SIP and MIP is due to the value of *ϵ* ^2^. MIP is defined as *ϵ* < 1, and therefore has a lower contribution to the excess coincidences than SIP, which explains the reduced sensitivity for MIP as compared to SIP processes. 

 In contrast, to the conditional spike frequency, *t* ^CPC^ evaluates the number of neurons that fire simultaneously with neuron *i*. We now present an analytical approximation of *t* ^CPC^ based on expected values following the line of argument introduced in [21]. For simplicity we focus on the SIP model. Let us first calculate the average complexity of a neuron that is part of the *M* neurons receiving injected events. The spikes of that neuron can be divided into spikes happening at times of occurrences of injected events and at times between the injected events. At injection times the total complexity is composed of the injected events of complexity *M* − 1 plus the complexity due to independent firing of the *N* − *M* independent neurons. Their complexity follows a binomial distribution [21] with probability *p* = *λ* *h* and complexities up to *N* − *M*, yielding on average a complexity of (*N* − *M*)*λ* *h*. At spike times when there is no injected event, coincidence patterns occur by chance. Their complexity is composed of chance coincidences of the *N* − *M* neurons with occurrence probability *λ* *h* as above, and chance coincidences of the *M* − 1 neurons with probability (*λ* − *λ*_*c*_)*h*. The latter component has a mean complexity (*M* − 1)(*λ* − *λ*_*c*_)*h*. Taken together this yields an average complexity sampled at spike times of an assembly neuron of

(11) $$\begin{array}{ll} & \frac{\lambda_{c}h}{\lambda h}\left[\left(M-1\right)+\left(N-M\right)\lambda h\right]\\ & \;\;\;+\left(1-\frac{\lambda_{c}h}{\lambda h}\right)\left[\left(M-1\right)\left(\lambda -\lambda_{c}\right)h+\left(N-M\right)\lambda h\right]\\ & \;\;=\left(M-1\right)p_{\text{obs}}+\left(N-M\right)\lambda h, \end{array}$$

with *p*_obs_ = *λ*_*c*_/*λ* + (1 − *λ*_*c*_/*λ*)(*λ* − *λ*_*c*_)*h*. In (3) for *t* ^CPC^ we compare the actual values to the average complexity considering all time bins (2). This average is given by the above expression with a different weighting of the two terms in square brackets

(12) $$\begin{array}{ll} & \lambda_{c}h\left[\left(M-1\right)+\left(N-M\right)\lambda h\right]\\ & \;\;\;+\left(1-\lambda_{c}h\right)\left[\left(M-1\right)\left(\lambda -\lambda_{c}\right)h+\left(N-M\right)\lambda h\right]\\ & \;\;=\left(M-1\right)p_{\text{tot}}+\left(N-M\right)\lambda h, \end{array}$$

with *p*_tot_ = *λ*_*c*_ *h* + (1 − *λ*_*c*_ *h*)(*λ* − *λ*_*c*_)*h*. Shuffling the spikes of neuron *i* ∈ {1,…, *M*} leads to a random sample of complexity instances, which by definition produces the same average complexity as that obtained by considering all time bins. Thus before shuffling spikes from neuron *i*, the expectation of the *t* ^CPC^ reads

(13) $$\begin{array}{cc} & \frac{\left[\left(M-1\right)p_{\text{obs}}+\left(N-M\right)\lambda h\right]-\left[\left(M-1\right)p_{\text{tot}}+\left(N-M\right)\lambda h\right]}{\left(M-1\right)p_{\text{tot}}+\left(N-M\right)\lambda h}\\ & \;\;=\frac{p_{\text{obs}}-p_{\text{tot}}}{\left(N/\left(M-1\right)\right)\lambda h+p_{\text{tot}}-\lambda h}=\frac{a}{\left(N/\left(M-1\right)\right)b-c}, \end{array}$$

with *a*, *b*, *c* > 0 and (*N*/(*M* − 1))*b* > *c*, while after the shuffling it is simply 0. The difference between the two reflects the performance of the test statistic and so the larger the difference is, the smaller the FN rate will be. As expected, this difference scales with the ratio of *M*/*N* and for fixed *N*, the larger *M* the larger the difference. 

 The performance of *t* ^CPC^ as a function of increasing coincidence rate can also be explained by an increase in *p*_obs_ − *p*_tot_. As for *t* ^CSF^, the fact that the performance improves for an enlarged population and fixed *M*/*N* ratio is explained by a reduction in the variance around the expected value, due to which the same expectation yields lower *P*-values and thus fewer false negatives. 

 All these arguments also hold for the test statistics for *α* > 1, however, due to the exponential weighting the contributions of the excess coincidences are even more emphasized.

### 4.3. Data Generated from a Synfire Chain Model

The second experiment to illustrate our technique is based on spike data from a simulated neural network containing an embedded synfire chain [3]. The network is based on the balanced random network proposed in [29] and contains 64 000 excitatory and 16 000 inhibitory neurons firing at 4.2 Hz. The neuron model implements current-based integrate-and-fire dynamics with postsynaptic currents (PSCs) represented as *α*-functions (for a detailed description see [30]). The model parameters are given in Table 1. Each neuron receives 6000 synaptic inputs. For the inhibitory neurons and excitatory neurons that are not part of the synfire chain, 3840 inputs are drawn randomly from the local excitatory population, 960 from the local inhibitory population, and the rest are considered to be connections from remote excitatory neurons, represented as independent Poisson processes firing at 14 Hz. The peak value of recurrent excitatory and inhibitory PSCs are 38.5 pA and −231 pA respectively. 

 The embedded chain consists of 20 pools of 100 excitatory neurons. Each neuron in a pool receives synaptic input from each neuron in the previous pool. To ensure robust propagation of synfire activity, the peak strength and synaptic delay of a feed-forward connection are set to 61 pA and 1.5 milliseconds respectively. Each chain neuron also receives 3740 inputs drawn randomly from the local excitatory population with a reduced peak strength of 37.56 pA, and inhibitory and remote connections as described earlier. Except for the feed-forward connections, synaptic delays are given by *d* · *h*, where *h* is the computational step size (0.1 milliseconds) and *d* is drawn from a uniform distribution between 1 and 29. 

 To activate synfire chains the first pool in the chain is stimulated with a large synchronous pulse at irregular intervals with a Poisson rate of 1 Hz (SFC). To provide a control case, the embedded synfire chain is not specifically stimulated (SFCu). A further control case is provided by a network that does not contain a synfire chain, that is, all neurons have the same input statistics (NoSFC). The respective synfire datasets are recordings of 100 second duration of *N* = 5000 neurons from these networks. *k* of these are randomly sampled from the 2000 synfire chain neurons, the other *N* − *k* excitatory neurons are randomly selected from the rest of the network. The sampling degree *k* was chosen as 10%, 25% and 100%.

All simulations were performed in NEST [31]; the simulation scripts are available from the authors on request.

### 4.4. Results for the Synfire Chain Data

#### 4.4.1. Embedded and Stimulated Synfire Chain

**Figure 5:**
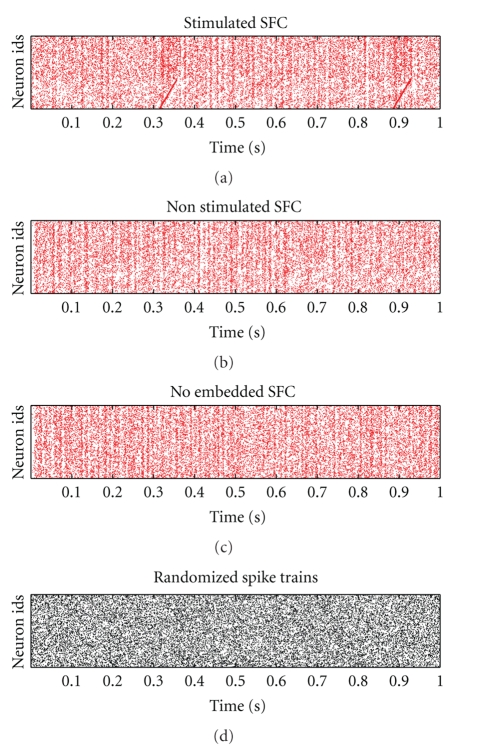
Dot displays of data from network simulations. (a) SFC: Network with embedded synfire chain (5000 neurons are displayed) the neurons in the lower part of the display (here 2000) are part of the synfire chain. Synfire runs can be observed after stimulation at times 0.31 seconds and 0.89 seconds. (b) SFCu: Network with the same connectivity as in (a) but without stimulation of the synfire chain. (c) NoSFC: Network with purely random connectivity (no embedded synfire chain). (d) Visualization of spike time randomization per neuron for the data shown in (a). The displays show time segments of 1 seconds duration.

One obvious feature of the simulated network data is the oscillatory nature of the activity (Figures 5(a)–5(c)) which is a characteristic feature of simulated balanced recurrent random networks [29, 32]. Global synchrony can be eliminated by disregarding the principle that neurons make exclusively excitatory or inhibitory outgoing connections [33]. However, in networks that do observe this principle, synchrony can be reduced but not eradicated by parameter choice. The key factors influencing the strength and frequency of the oscillations are the comparative strengths of excitatory and inhibitory synapses, the strength of the external stimulation [29] and the distribution of the synaptic delays [34]. In extreme these factors lead to fully synchronous activity. Here we chose parameters that result in asynchronous irregular activity, that is, there is little synchrony between the neurons and each spike train is irregular. Nonetheless, residual oscillatory structure can be observed even in the absence of embedded structure (Figure 5(c)). 

**Figure 6:**
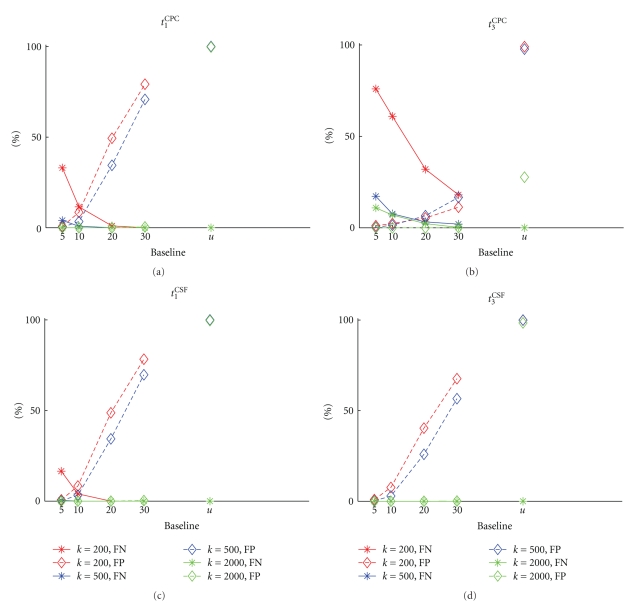
FN and FP for data of a SFC network with stimulated SFC. All panels show FP (diamonds and dashed lines) and FN rates (stars and solid lines) as a function of baseline level for the spike time randomization (*x*-axis). The curves of different colors in a panel represent different sample sizes of the SFC (*k* = 200,500,2000) within a total of *N* = 5000 observed and analyzed neurons. Each of the different panels displays the results for a specific test statistic ((a): *t*_1_^CPC^, (b): *t*_3_^CPC^, (c): *t*_1_^CSF^, (d): *t*_3_^CSF^).

**Figure 7:**
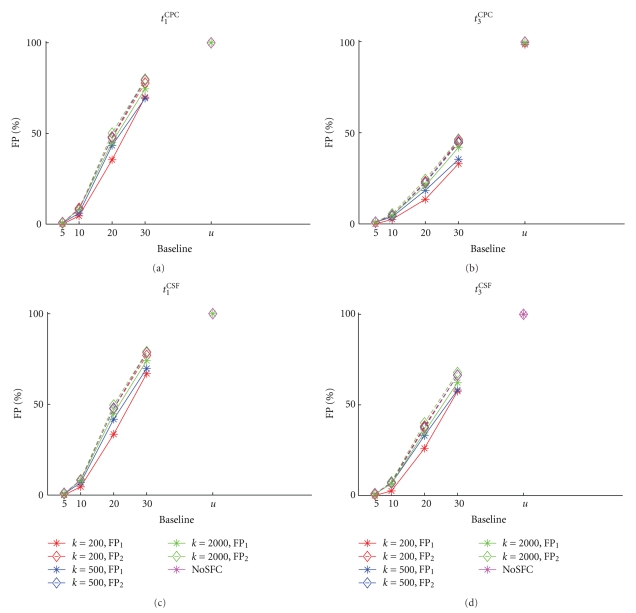
FP rates of SFCu and NoSFC data as a function of the base line level of the spike time randomization (*x*-axis). Each panel displays the results for a different test statistic ((a): *t*_1_^CPC^, (b): *t*_3_^CPC^, (c): *t*_1_^CSF^, (d): *t*_3_^CSF^). The curves within each panel show the percentage of FPs for different sampling sizes from SFCu (*k* = 200, 500, 2000, of a total of *N* = 5000 analyzed neurons). False positives are distinguished between FPs from neurons that are members of the embedded chain (FP_1_, stars and solid lines) and neurons that are part of the rest of the network (FP_2_, diamonds and dashed lines). The magenta curves show the FP rate for *N* = 5000 randomly selected neurons from a network without an embedded chain (NoSFC).

 Irrespective of the origin of these oscillations, the generation of the surrogates have to be carefully chosen to avoid false outcomes. Surrogates with uniform spike shuffling clearly destroy the oscillatory feature (Figure 5(d)), which leads to false-positive detections in the data with stimulated SFC (Figure 6, “*u*”) as well as for nonstimulated SFC or even when no SFC is embedded (see Figure 7, “*u*”). Thus a spike shuffling approach that accounts for the coherent rate oscillations of the neurons as introduced in Section 2.3 seems to be more appropriate. To this end we consider the population histogram as an estimate for the inhomogeneous weighting of shuffle times including an additional baseline. If neurons were not coherently oscillating, the estimator of the population histogram would not be appropriate. 

 The level of the baseline is a free parameter and has to be chosen according to the degree of modulation of the population firing rate. Since we know which neurons are members of the synfire chain we can systematically vary the baseline level and evaluate the resulting FP rate. Figure 6 illustrates that the larger the baseline, the higher the FP rate (diamonds and dashed lines). This effect is to a large extent independent of the chosen test statistic (panels a–d), but differences are more prominent dependent on the sample size *k* of the neurons from the total of 2000 members of the synfire chain. When all synfire chain neurons are sampled (*k* = 2000, green diamonds and dashed lines), the FP rate abruptly changes from 0% FP to a high level. For sample sizes smaller than 2000, the increase of FP with baseline is more gradual. In summary, a baseline level of 5 leads to a 0% FP rate, independently of the test statistic or sample size. 

 In contrast, the FN rate (Figure 6, filled circles and solid lines) depends strongly on the test statistic used. The FN rate typically decreases with baseline level, and the smaller the sample size *k* the higher the FN rate. However, for test statistic *t*_3_^CSF^ no FNs occur (Figure 6(d)), even for small sample sizes. Thus a choice of *t*_3_^CSF^ and surrogates with a baseline level of 5 lead to perfect detection of the member neurons of the synfire chain. Different baseline levels may be tolerated at the price of a higher FP rate if further analysis emphasizing higher-order analysis of the data is performed.

#### 4.4.2. Controls

Since data from the network containing a nonstimulated synfire chain and the network with no embedded synfire chain also exhibit the oscillatory firing rate coherent across neurons, we tested the FP rate as a function of the baseline level (Figure 7) for these control datasets as well. The FP rates behave indistinguishably from the data with stimulated synfire chains (Figure 6) and do not show any dependence on the test statistic chosen. This holds true for FPs for the subset of *k* neurons selected from the synfire neurons and for neurons from the rest of the network (compare FP_1_ and FP_2_ in Figure 7). Data from the network with no embedded synfire chain show the same results. This confirms that the oscillatory structure in the data, which is not related to the synfire activation, is responsible for increasing FP rates for increasing homogeneity of the surrogates. The strongest generator of FPs is uniform shuffling.

### 4.5. Real World Spike Data

After having tested and calibrated our analysis approach, we finally demonstrate its applicability to neuronal data. We chose to apply it to a dataset recorded from cat visual cortex that we have extensively studied with other analysis methods, so that we can compare the results.

#### 4.5.1. Experimental Procedures

Parallel spike recordings were obtained by a 10 × 10 electrode grid (Utah electrode array, Bionic Technologies, Inc., Salt Lake City, Utah, USA) covering an area of 3.6 mm × 3.6 mm of cat visual cortex [35]. Data were recorded from area 17 of anesthetized cat under full flash treatment. Data were recorded either under spontaneous condition, that is, without visual stimulation, or with a full flash stimulus. The intensity of the latter was changed alternating from high intensity to low intensity, each presented for 0.51 seconds and 2.7 seconds, respectively. Experiments were performed under animal care and experimental guidelines that conformed to those set by the (National Institute of Health) NIH. Animals were obtained from the University of Utah Animal Resource Center. Description of the animal preparation, maintenance, and surgery procedures is fully described in [35, 36].

#### 4.5.2. Previous Results

In [18] we analyzed the spiking activities recorded from the grid of electrodes for pairwise correlations. High intensity (HI) and low intensity (LI) epochs were separated into different datasets and analyzed separately. For simplicity we restricted ourselves to the evaluation of the multiple-unit activities (MUAs), which here were composed of a mixture of typically 3 neurons. The cut-off for considering a MUA for the correlation analysis was a minimal firing rate of 1 Hz. Some electrodes were broken. This left us with 85 parallel MUA spike trains for LI and HI. From these we computed the cross-correlations (CCH) [37] for all possible MUA pairs. To evaluate the significance of the correlation, we used a boot-strap method (spike dithering with a dither width of ±35 milliseconds [38]) that accounts for the changes in firing rate of the neurons. Spike correlation between two MUAs was considered significant if the original CCH exceeded the mean of the surrogate CCHs (100 surrogates, smoothed with a boxcar kernel of width 10 milliseconds) by 2*σ* (i.e., a significance level of 5%). As a result 78/3402 pairs for HI and 203/3402 pairs for LI were significantly correlated. 

**Figure 8:**
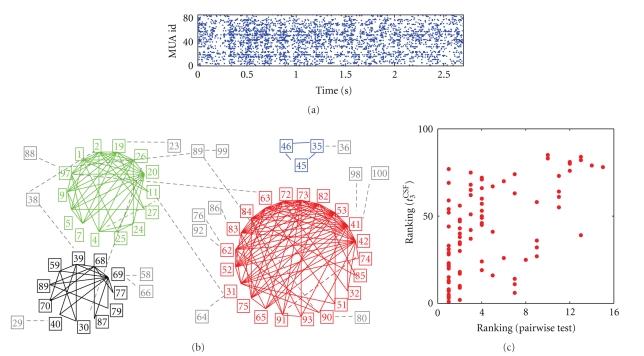
Analysis of parallel spike trains from cat visual cortex. (a) Dot display of 85 multiunit spiking activities recorded simultaneously during low intensity full flash stimulation. The dot display shows the 3rd trial of low intensity stimulation lasting for 2.7 seconds. (b) Results of pairwise correlation analysis and subsequent clustering of groups of intracorrelated MUAs of the same data under consideration of all 16 trials (Figure modified from [18]). (c) Comparison of the results of the pairwise correlation analysis and our identification of potential assembly member neurons using *t*_3_^CSF^. Surrogates are generated by trial shuffling. Each dot in the scatter display shows the rank of each MUA according to the number of involvements in significant pairwise correlations (*x*-axis; rank of 1 corresponds to the smallest number) and the ranking according to the value of the test statistic *t*_3_^CSF^ with baseline *u* (*y*-axis; rank 1 corresponds to the lowest value).

 We noticed that individual MUAs were often not only correlated with a single other MUA, but typically with more than 2 (up to 15). This led us to a graph theoretical analysis to discover whether groups of intracorrelated MUAs can be identified. In a first step we identified cliques with greater than 2 members, that is, groups of MUAs that are all-to-all mutually correlated. Since these turned out to exist abundantly and with overlapping membership, we further grouped them using the criteria of a minimal overlap of one member. Interestingly, as a result the graph decomposed into a small number of completely disjoint subgraphs (see Figure 8(b)). These clusters of highly intracorrelated neurons also clustered in cortical space with a similar space constant to orientation tuning maps found by optical imaging [39], strongly suggesting a relationship between them.

#### 4.5.3. Comparison of Analysis Results

We reanalyze the neuronal data with the approach presented here to identify potential assembly neurons. The dot display of the simultaneous spike trains of the cat visual cortex data (Figure 8(a)) indicate nonstationarities in the firing rates that appear in a oscillatory and covarying fashion similar to the simulated network data. Therefore, we analyze the neuronal data in the same way as the network data by using weighted spike time shuffling. The comparison of the various test statistics on the network data (Section 4.4) revealed similar behavior for all test statistics but *t*_3_^CSF^ showed best performance (very low FP rate and low FN rate). We therefore analyze our neuronal data with *t*_3_^CSF^ and also vary the baseline in the same range as in the former analysis. 

 The correlation structures in the real-world data are unknown to us, as we do not know the underlying neural processes and connectivity. However, we have already studied the chosen dataset extensively using other analysis methods, and it is of interest to determine whether different analysis strategies lead to consistent results. Here we aim to identify whether individual MUAs are members of groups of neurons that exhibit synchronous spiking activity, whereas in the former study we identified groups of MUAs by significant pairwise correlation and their high degree of multiple involvements in correlation. 

**Figure 9:**
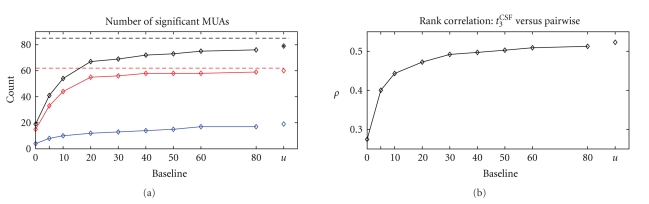
Comparison of results of different analysis approaches. (a) The pairwise analysis revealed 62 MUAs (red dashed line) out of 85 MUAs (black dashed line) to be significantly involved in pairwise correlations. The analysis with the test statistic *t*_3_^CSF^ using weighted spike time shuffling with increasing baseline detects an increasing number of MUAs (black curve). The number of MUAs detected by both methods is indicated by the red curve. The blue curve indicates the number of MUAs detected by *t*_3_^CSF^ but not by the pairwise analysis, that is, are not involved in any significant pairwise correlation. (b) Rank correlation *ρ* (Spearman) of the rankings of two approaches (cf. Figure 8(c) for *t*_3_^CSF^ with baseline *u*).

Figure 9 shows a comparison of the two approaches. The black curve shows the total number of MUAs detected by *t*_3_^CSF^, which increases with increasing baseline. For a baseline between 10 and 20 this number surpasses the number detected by the former pairwise analysis (red dashed line). The number of MUAs detected in both analyses (red curve) also increases with the baseline and approaches the total number detected by the pairwise analysis for high baselines, that is, highest uniformity. The differences between the total detected numbers is represented by the blue curve, showing MUAs that are detected by *t*_3_^CSF^ but that are not involved in any significant pairwise correlation. 

 The analysis of the network data using *t*_3_^CSF^ (Figure 6(d)) demonstrates an increase of false-positives for increasing baseline. The reason is the increasing destruction of the rate modulation and thus of the coincident events occurring by chance. However, since the chance coincidences are still present in the original data they may be detected as significant outcomes. The more uniform the shuffling, the more destruction and the greater the number of FPs. In addition, this effect becomes stronger as the modulation depth of the rate profile increases. The oscillatory nature of the neuronal data analyzed here, however, appears with less modulatory depth and lower oscillation frequency (approx. 4 Hz) than the network data. Thus, it is likely that the optimal baseline for the spike time shuffling is different for this data. In addition, the rate modulation appears to be incoherent across the neurons. However, spike time shuffling according to the population histogram estimate assumes homogeneity of firing rates across the neurons. Thus, it is very likely that some MUAs experience a spike time shuffling that is more strongly modulated than their original rate profile, while for others it is too weakly modulated. 

 In the light of these considerations, the comparison of the analysis results may be interpreted as follows. The overlap of the detected MUAs for the two analyses (red curve in Figure 9(a)) increases quickly for small baselines, and from a baseline of about 20 slowly converges to the number detected by the pairwise analysis. One may speculate that MUAs with less modulated rate are detected only with higher baselines, since for lower baseline their chance coincidence level is overestimated. This could explain why two MUAs are not detected as significant by the test statistic for any baseline although the pairwise correlation analysis revealed that they are both involved in 6 pairwise correlations. Similarly, one may speculate that the additionally detected MUAs by *t*_3_^CSF^ (blue curve in Figure 9(a)) reflect MUAs which are not involved in coincident events but have a strong rate modulation which is underestimated by the population rate estimate and thus leads to wrongly detected MUAs. The previous pairwise analysis took the individual rate profiles into account by generating the surrogates based on spike dithering and therefore did not detect those MUAs. Clearly, these considerations still have to be tested in detail and will be the subject of future studies. MUAs not detected by either analysis are either not involved in any pairwise correlation or are only involved in a pairwise correlation with a MUA that is not part of a cluster. 

 Another aspect for the comparison of the two analysis results is that the pairwise correlation analysis revealed multiple involvements of individual MUAs in correlated pairs, and that correlated pairs form clusters of highly intracorrelated members. This may hint at the existence of higher-order correlation between the neurons in a cluster, or at involvement in many, strongly overlapping assemblies. This could not be decided on the basis of the previous analysis, but requires additional higher-order analysis. Such methods are currently being developed [40, 41] and make use of similar measures to those used here for the test statistics. In the context of the analysis of the stochastic model data (Section 4.2.2) we showed that the larger the assembly, that is, the more neurons involved in synchronous events, the better the detection of the neurons of the assembly neurons (Figure 3). This is also reflected in the definition of *t*_3_^CSF^ (7): the test statistic increases with increasing number of excess synchrony the neuron is involved in. We further explored the relation of the two analyses by comparing the number of involvements of the individual MUAs in significant pairwise correlations to the strength of the test statistic. We quantify the relationship of these measures by the correlations of their ranks. The ranking of a MUA according to the *t*_3_^CSF^ analysis is determined by the actual value of the test statistic normalized to the width of the surrogate distribution represented by the difference between its 0.99 and 0.01 quantiles. Note that the *P*-value cannot serve for the ranking since there are not enough states. The ranking of a MUA according to the pairwise analysis is given by the number of pairwise correlations it is involved in. The relationship between the two rankings is shown in the form of a scatter plot in Figure 8(c). Clearly, the rankings by the two analyses are correlated, that is, the test statistic is greater for MUAs that are involved in high numbers of pairwise correlations. The quantification of their rank order correlation (Spearman) is shown in Figure 9(b) as a function of the baseline of the surrogates. The curve shows a very similar behavior to the graphs representing the number of MUAs detected. This indicates that MUAs involved in many correlations are more likely to be detected by the test statistic, however involvement in higher-order correlations cannot be concluded.

## 5. Discussion

We presented two simple test statistics, each parameterized with two different values (*α* = 1 and *α* = 3), to identify neurons that are involved in assemblies. Both of these statistics test for a given neuron whether it is involved in coincident spike events more often than can be expected by chance. To do so, *t* ^CPC^ analyses the coincidence complexities of the parallel spike trains, and *t* ^CSF^ aggregates pairwise frequency comparisons. In order to assess their performance, we applied these statistics, using a spike shuffling approach, to massively parallel spike trains (either 100, 1000, or 5000) generated by stochastic models and network simulations, and to real-world neural spike data. The data generated by the stochastic model served to test and calibrate the test statistics, because these models enable us to define different spike correlation structures, rate of correlation occurrence and number of processes involved in the correlation. Our various test statistics are very sensitive with a high detection rate and a low false-positive rate that corresponds to the applied significance level. The false negative rate is high for very low coincidence rates or very small percentage of neurons involved in the correlation, for *N* = 100 and *N* = 1000 respectively, but drops drastically for larger values for either of the two parameters. The statistic *t*_3_^CSF^, that is, *t*_*α*_^CSF^ with *α* = 3, behaves slightly better for the detection of assembly members in the simulated data. We conclude that our approach could prove a reliable tool for the detection of potential assembly members. 

 The data generated by network simulations exhibits additional properties that are not present in the stochastic model data. The firing rates of the neurons appear to be nonstationary in time, with a high oscillation frequency (about 100 Hz). As a consequence, the way of generating the surrogates used for evaluating the significance of our measures had to be adjusted. Our results clearly demonstrate that a uniform spike shuffling is a strong generator of false-positives. If instead the spike shuffling is performed according to an estimator that is based on the population firing rate, the false-positive rate can be reduced to close to zero if the modulation of the shuffling probabilities as a function of time is high (i.e., low baseline). The control data, which do not contain any activated synfire chains, also exhibit large FP rates for increasing baseline. Obviously, the coherent rate oscillations generate a high amount of synchronous events, which, if ignored by for example, uniform shuffling, would be interpreted as the existence of precise spike synchrony. 

 Interestingly, the false negative rate is close to zero for *t*_3_^CSF^, even for smaller numbers of neurons sampled from the embedded synfire chain. Although a sampling of 200 out of 2000 neurons sounds large as a percentage, the number of neurons sampled from an individual group of the synfire chain, in which synchronous spike events occur, is rather small (200/20 = 10), a number comparable to the assembly size *M* studied for the stochastic model data. The finding that we can accurately detect assembly neurons even when sparsely sampled and in the presence of significant global synchrony suggests that our technique will be helpful in analyzing real-world data, which often exhibits oscillations and other nonstationarities. 

 After having tested our approach in simulated data we applied it to neuronal parallel spike data from cat visual cortex. We chose this particular data because we have already studied the chosen dataset extensively using other analysis methods, in particular pairwise correlation analysis with subsequent clustering. Thus, we know that the data contain correlated spiking, and that there exist groups of neurons that are highly intracorrelated. The data exhibits a similar oscillatory structure in the population activity to the network simulations. Thus we also applied weighted spike time shuffling for generation of the surrogates, and varied the baseline as for the network data. For increasing baseline, our simple test statistics detect an increasing number of MUAs that had previously been detected in the pairwise analysis. For the largest baseline it attains an almost perfect detection of all the MUAs that are involved in significant pairwise analysis. The detected MUAs are mostly involved in more than one significant pairwise correlation. Those which are not detected are mostly involved in none, or in just one correlation with a MUA which is not a member of a cluster. These results are also reflected by the strong correlation between the strength of the test statistics and the number of involvements in pairwise correlations, and may hint at the presence of higher-order synchrony within MUAs of the same clusters. Indeed, preliminary results from higher-order analysis based on accretion [42, 43] performed on the same dataset revealed that the identified clusters reflect higher-order correlations between MUAs of a cluster. In conclusion, we are confident that our test statistic picks up the presence of larger groups of correlated neurons. 

 However, as the baseline increases there is also an increasing number of MUAs that are detected by the test statistic but are not involved in any pairwise correlation. It may well be that these are wrongly detected due to inappropriate surrogates for the respective MUAs. Although the population firing rate seemingly modulates in oscillatory fashion, one can clearly see that the MUAs exhibit inhomogeneities in the firing rate levels and their modulation depths. However, weighting of the spike time shuffle according to the population rate is based on an average across the MUAs assuming homogeneity. As a result, the estimate based on the population rate average can either under- or overestimate the firing rate modulation of a MUA. The former tends to lead to false-positive outcomes, and the latter to false negatives. Thus one could speculate that the MUAs detected by the test statistics but not by the pairwise analysis are detected due to an inappropriate baseline of the surrogate. Spike time shuffling adjusted to the individual MUAs and their firing rate time course seems to be more appropriate. Possible candidates could be dithering of individual spike times [38] or of the whole spike trains [44, 45]. The choice of the proper surrogate and its impact on the performance of the test statistic will be the subject of further research. 

 Our approaches are a valuable addition to methods that provide information on the * presence* of higher-order correlation in data, but do not identify the individual neurons involved [41]. However, their most important use is as a preprocessing technique before applying computationally more expensive analyses. Existent methods for correlation analysis [13, 14, 40, 46] and in particular higher-order correlation analysis of such high-dimensional data are very time consuming and often not applicable due to the immense memory consumption or computation time. The test methods we presented here are intended to reduce large datasets to the relevant neurons, thus reducing computation time considerably for the further analysis steps. Therefore, it is crucial to achieve a low false negative rate so that significantly correlated neurons will not be missed. On the other hand, false-positives can largely be tolerated, in particular if subsequent analysis focuses on higher-order analysis. Our results show that our test statistics provide the required high sensitivity and thus serve as an effective preprocessing method. Moreover, our methods are efficient, fast, trivially parallelizable and can easily digest recordings of an order of thousands of parallel processes. To illustrate this, for the cat visual cortex data analyzed in Section 4.5.3, each MUA could be shuffled 5000 times in less than one second on a standard desktop computer. In other words, each data point in Figure 9 could be calculated in 80 seconds. Indeed, our methods are so fast that they would even be appropriate for determining relevant neurons in a sliding window over a long recording period, thus enabling dynamical changes of the correlation to be recovered.

## Figures and Tables

**Table 1:** Neuron model parameters.

Parameter	Value
Membrane time constant	10 milliseconds
Membrane capacitance	250 pF
Threshold	20 mV
Refractory period	0.5 milliseconds
rise time of PSC	0.3258 milliseconds
